# Systematic Characteristics of Fucoidan: Intriguing Features for New Pharmacological Interventions

**DOI:** 10.3390/ijms252111771

**Published:** 2024-11-01

**Authors:** Seungjin Jeong, Seokmin Lee, Geumbin Lee, Jimin Hyun, Bomi Ryu

**Affiliations:** 1 Department of Food Science Nutrition, Pukyong National University, Busan 48513, Republic of Korea; wtw3737@pukyong.ac.kr (S.J.); tjrals0300@pukyong.ac.kr (S.L.); lijinbin369@pukyong.ac.kr (G.L.); 2 Department of Smart Green Technology Engineering, Pukyong National University, Busan 48513, Republic of Korea

**Keywords:** brown algae, fucoidan, monosaccharide, molecular weight, structure, sulfation, biological activity

## Abstract

Fucoidan, a sulfated polysaccharide found primarily in brown algae, is known for exhibiting various biological activities, many of which have been attributed to its sulfate content. However, recent advancements in techniques for analyzing polysaccharide structures have highlighted that not only the sulfate groups but also the composition, molecular weight, and structures of the polysaccharides and their monomers play a crucial role in modulating biological effects. This review comprehensively provides the monosaccharide composition, degree of sulfation, molecular weight distribution, and linkage of glycosidic bonds of fucoidan, focusing on the diversity of its biological activities based on various characteristics. The implications of these findings for future applications and potential therapeutic uses of fucoidan are also discussed.

## 1. Introduction

Polysaccharides are complex carbohydrates composed of long chains of monosaccharide units linked together by glycosidic bonds. The structural complexity of these carbohydrates renders polysaccharides such as carrageenan, lignin, and chitosan therapeutically valuable. Among the polysaccharides with therapeutic potential, fucoidan—a marine polysaccharide derived from algae and containing fucose—exhibits a broad range of biological activities [1,2,3,4,5]. It is well known that the sulfate group content is important in determining the biological activity of algae-derived fucoidans [6,7,8]. However, recent technical advancements in the analysis of structural features such as its monosaccharide composition, degree of sulfation, molecular weight distribution, and glycosidic bond linkages have provided a more nuanced understanding of how these characteristics influence fucoidan’s biological functions [9,10,11].

With improvements in analytical techniques, it has become possible to more accurately characterize the structural properties of fucoidan, a complex marine sulfated biopolysaccharides with heterogeneous chemical structures, which were previously difficult to elucidate [12,13]. As a result, there has been a surge in studies investigating the relationship between fucoidan’s physicochemical characteristics and bioactivity [14]. While early research focused primarily on the correlation between the degree of sulfation and its bioactivity, more recent studies have examined the impact of other structural features, including monosaccharide composition, molecular weight distribution, and glycosidic bond linkages [15,16,17]. This review presents the activities of fucoidan based on its characteristics, focusing on its biological activities, including its anticoagulant, antitumor, anticancer, anti-inflammatory, antioxidant, immunomodulatory, hypocholesterolemic, antibacterial, and anti-SARS-CoV-2 effects.

Emerging evidence suggests that the monosaccharide ratio and types of monosaccharides, such as fucose, galactose, and mannose, play a crucial role in modulating both the efficacy and mechanisms of action of fucoidan’s biological effects [18].

This review aims to synthesize recent findings on how the structural composition of fucoidan influences its biological activities, providing a comprehensive overview of how these functional properties are determined (Table S1). Additionally, it explores how these insights can inform future research directions and guide the development of potential therapeutic applications of fucoidan in various fields.

## 2. The Role of Monosaccharides of Fucoidan in Bioactivity

Fucoidan, a polysaccharide derived from algae, is notable for its high fucose content, a monosaccharide that significantly contributes to its bioactivity [19,20]. The relationship between fucose content and the biological activities of fucoidan—such as its antioxidant, anticoagulant, antiviral, and anti-inflammatory effects—has been well established [21,22,23,24,25,26]. Research indicates that fucoidan with a higher fucose content often exhibits enhanced biological efficacy [22,27]. For instance, fucoidans with elevated fucose concentrations tend to exhibit stronger anticoagulant and anti-inflammatory properties, likely due to the unique sulfation patterns associated with fucose residues [28,29,30]. However, while fucose is a key component, its content alone does not solely determine the bioactivity of the polysaccharide.

Additionally, other monosaccharides present in fucoidan, such as galactose, mannose, glucose, and xylose, play significant roles in modulating its overall bioactivity [31,32,33]. These sugars influence the molecular weight, structural configuration, and functional properties of fucoidan, thereby affecting its interactions with biological targets [34,35,36]. For instance, mannose and galactose have been shown to enhance the antiviral and immunomodulatory effects of fucoidan, while glucose and xylose can impact its antioxidant capacity [37,38,39]. Thus, while fucose is crucial for fucoidan’s activity, the specific composition and ratio of other monosaccharides are equally vital for optimizing its therapeutic potential (Table 1).

Research has demonstrated that the presence of monosaccharides in fucoidan can significantly influence its bioactivity. For example, Rashed et al. (2021) identified a fucoidan fraction containing fucose, glucose, galactose, and mannose that exhibited high antioxidant capacity and antisteatotic action [40]. Ptak et al. (2021) suggested that glucose content may affect bioactivity, likely due to the presence of laminarin in the extract [41]. Furthermore, Dobrinčić et al. (2021) noted that extraction conditions could alter the content of various monosaccharides and sulfate groups, thereby influencing fucoidan’s properties [42]. The arrangement of these monosaccharides, together with the degree of sulfation, plays a crucial role in determining fucoidan’s overall bioactivity, emphasizing the importance of both sugar composition and sulfation patterns in its therapeutic potential.

The biological effects of fucoidan are closely tied to its specific monosaccharide composition, which varies significantly across different algae species. For instance, Bilan et al. (2017) extracted fucoidan from *Sargassum aquifolium* (Phaeophyceae, Fucales), predominantly composed of fucose, galactose, mannose, glucuronic acid, and xylose, which have demonstrated robust anticoagulant and anticancer effects in vitro, as evidenced by their activity against HePG2, A549, and HBL-100 cell lines [43]. Similarly, polysaccharides from *Undaria pinnatifida* (Phaeophyceae, Laminariales), containing fucose, glucuronic acid, galactose, and mannose, exhibit potent antioxidant properties in zebrafish models at concentrations starting from 0.625 mM, while also showing efficacy in attenuating SARS-CoV-2 infection in hamster models at dosages of 100 and 200 mg/kg body weight [44,45]. The polysaccharides from *Fucus vesiculosus* (Phaeophyceae, Fucales), which consist of fucose, xylose, galactose, and uronic acids, have been shown to suppress inflammatory responses in RAW 264.7 macrophage cells and demonstrate significant antioxidant activity in zebrafish models at a concentration of 0.625 mM [45,46]. Additionally, *Sargassum fusiforme* (Phaeophyceae, Fucales) polysaccharides, rich in fucose, galactose, and mannose, have been reported to exhibit anti-inflammatory effects in both RAW 264.7 macrophages and zebrafish embryos at concentrations of 25, 50, and 100 μg/mL [47].

Moreover, the polysaccharides of *Fucus serratus* (Phaeophyceae, Fucales), containing fucose, galactose, glucuronic acid, and xylose, and *Fucus distichus* subsp. *evanescens* (formerly *Fucus evanescens*), containing fucose, galactose, and xylose, have also shown remarkable potential in promoting bone formation and vascularization, as demonstrated in human outgrowth endothelial cells (OECs) and mesenchymal stem cells (MSCs) at a concentration of 100 μg/mL [48]. In terms of immunomodulatory and antiviral activity, *Saccharina latissima* (Phaeophyceae, Laminariales) polysaccharides, composed of fucose, xylose, galactose, mannose, glucuronic acid, and uronic acids, have been found to stimulate lymphocyte proliferation in spleen cells derived from BALB/c mice at concentrations ranging from 25 to 250 μg/mL [49,50]. Furthermore, *Stoechospermum polypodioides* (formerly *Stoechospermum marginatum*) (Phaeophyceae, Dictyotales) polysaccharides, composed of fucose, xylose, mannose, galactose, glucose, and galacturonic acid, have shown potent antiviral activity against a range of viruses in Vero cells, with an effective concentration (EC50) of 3.5 ± 0.63 μg/mL [51]. Polysaccharides from *Cladosiphon okamuranus* (Phaeophyceae, Ectocarpales), characterized by a high content of fucose and uronic acid, have demonstrated effectiveness in mitigating atopic dermatitis symptoms through immunomodulation in BALB/c mice models [52].

Furthermore, *Saccharina japonica* (formerly *Laminaria japonica*) (Phaeophyceae, Laminariales) polysaccharides, comprising fucose, mannose, and glucose, have been reported to possess antibacterial properties and anti-SARS-CoV-2 activity in studies involving various bacterial strains and viral infection models, with effective concentrations ranging from 6.25 to 50 μg/mL [53]. Collectively, the diversity in monosaccharide composition among these polysaccharides underpins their wide-ranging bioactivities, offering a compelling basis for their application in therapeutic interventions. However, to fully exploit the bioactive potential of these algae-derived polysaccharides, further research is needed to refine the methods for extraction, purification, and characterization, thereby enabling more precise control over their composition and enhancing their therapeutic efficacy across various biomedical domains.

The bioactivity of fucoidan is not solely dependent on its fucose content; it is the intricate interplay between fucose and other monosaccharides that determines its full range of biological effects. Understanding these relationships is crucial for optimizing the use of fucoidan in various biomedical applications. While fucose is an essential component of fucoidan, its content alone does not dictate bioactivity. The sulfate content, the presence of other monosaccharides, and the overall structural characteristics of the polysaccharide all contribute to its biological activities. This complex interplay highlights the importance of considering multiple factors when evaluating the potential therapeutic applications of fucoidan.

**Table 1:** Bioactivity according to monosaccharide composition of brown algae fucoidan.

Algae Source	Monosaccharide Composition	Bioactivity	Experimental Methodologies	Experiment Model	Concentration	Reference
*Sargassum aquifolium*	Fuccose, galactose, mannose, glucuronic acid, xylose	Anticoagulant and antitumor activities	In vitro	Human cancer cell lines HepG2 (hepatocellular carcinoma), LU-1 (lung adenocarcinoma), and RD (rhabdomiosarcoma)	ND	[43]
*Sargassum plagiophyllum*	Fucose, galactose, xylose, mannose	Anticancer activity	In vitro	HepG2, A549, and HBL-100	ND	[54]
*Sargassum horneri*	Fucose, galactose, mannose, xylose, rhamnose	Anti-inflammatory activity	In vitro	RAW 264.7 cells	EC50 (μg/mL): 87.12	[22]
	Fucose, galactose, mannose, glucuronic acid	Antitumor	In vitro	The DLD-1 (ATCC # CCL-221™) human colon carcinoma cell line	200 μg/mL	[55]
*Undaria pinnatifida*	Fucose, glucuronic acid, galactose, mannose	Antioxidant activities	In vivo	Zebrafish	0.625 mM	[45]
	Fucose, glucose, galactose	Anticancer activity	In vivo	Sprague Dawley rats	100, 200, and 300 mg/kg of body weight	[56]
	Fucose, glucuronic acid, galactose, mannose	Attenuation of SARS-CoV-2 infection	In vitro In vivo	Caco-2-Nint cells, a producer cell line expressing the SARS-CoV-2 N protein via lentiviral transduction	7.8, 15.6, 31.3, 62.5, 125, 500, and 1000 μg/mL	[44]
*Ascophyllum nodosum*	Fucose, galactose, mannose, glucuronic acid, uronic acid			Four-week-old female specific-pathogen-free (SPF) Syrian hamsters	Orally gavaged with high dose (Hd; 200 mg/day/kg body weight) or low dose (Ld; 100 mg/day/kg body weight)	
*Dictyopteris divaricata*	Fucose, xylose, mannose, glucose, galactose	Antioxidant and immunomodulatory activities	In vivo	RAW 264.7 murine macrophages	ND	[57]
*Sargassum crassifolium*	Fucose, galactose	Immunomodulatory activity	In vitro	Bone marrow cells from C3H/HeJ female mice	3 μg/mL	[58]
*Stoechospermum polypodioides*	Fucose, xylose, mannose, galactose, glucose, galacturonic acid	Antiviral activity	In vitro	Vero cells by a virus plaque reduction assay	EC50 (μg/mL): 3.55 ± 0.63	[51]
*Sargassum polycystum*	Fucose, xylose, mannose, galactose, glucose, rhamnose	Antioxidant activity, anticancer activity	In vitro	MCF-7 cells	25, 50, 75, 100, 125, and 150 μg/mL	[59]
*Sargassum siliquosum*	Fucose, xylose, mannose, galactose, glucose, rhamnose	Antioxidant activity, anti-inflammatory activity	In vitro	RAW 264.7 cell	0.25–1 μg/mL	[15]
*Fucus serratus*	Fucose, galactose, glucuronic acid, xylose	Bone formation and vascularization	In vitro	Human outgrowth endothelial cells (OECs), mesenchymal stem cells (MSCs)	100 μg/mL	[48]
*Fucus distichus* subsp. *evanescens*	Fucose, xylose, galactose					
	Fucose, xylose, mannose, galactose, glucose, glucuronic acid	Anticancer activity	In vitro	The SK-MEL-5 (ATCC # HTB-70), SK-MEL-28 (ATCC # HTB-72) human malignant melanoma cell lines	100–400 μg/mL	[60]
*Saccharina latissima*	Fucose, xylose, galactose, mannose, glucuronic acid, uronic acid	Immunostimulatory, hypocholesterolemic activities	In vitro	Lymphocyte Stimulatory Activity (spleen cell of BALB/c mice) Assessment of Hypocholesterolemic Effect (in vitro intestinal model)	25, 100, and 250 μg/mL	[49,50]
*Cladosiphon okamuranus*	Fucose, uronic acid	Alleviates atopic dermatitis symptoms through immunomodulation	In vitro In vivo	RAW 264.7 cells; atopic dermatitis (AD) model_Male BALB/c mice aged 6 weeks old	31.25, 62.5, 125, 250, 500, and 1000 μg/mL 100 mg of the cream	[52]
*Sargassum fusiforme*	Fucose, galactose, mannose	Anti-inflammatory	In vitro In vivo	RAW 264.7 macrophages; zebrafish embryo	25, 50, and 100 μg/mL	[47]
*Macrocystis pyrifera*	Fucose, Xylose, Glucuronic acid	Antioxidant activities	In vivo	Zebrafish	0.625 mM	[45]
*Padina boergesenii*	Fucose, galactose, glucose, xylose	Antioxidant and anticancer	In vitro	Human cervical carcinoma cells (HeLa cell line)	20, 40, and 60 μg/mL	[61]
*Fucus vesiculosus*	Fucose, xylose, galactose, mannose	Antioxidant activities	In vivo	Zebrafish	0.625 mM	[45]
	Fucose, xylose, galactose, uronic acids	Inhibition of inflammatory response	In vitro	RAW 264.7 macrophages	0.1 μg/mL	[46]
*Saccharina japonica*	Fucose, mannose, glucose	Antibacterial activity and anti SARS-CoV-2	In vitro	Bacteria including Staphylococcus aureus ATCC6538, Listeria monocytogenes ATCC19115, Escherichia coli ATCC25922, Shigella flexneri CMCC51574, Salmonella typhimurium ATCC14028, and Vibrio parahaemolyticus CGMCC1.1614	0, 6.25, 12.5, 25, and 50 μg/mL	[53]
*Ishige okamurae*	Fucose, galactose, glucose, xylose	Effect on recovery from immunosuppression	In vivo	BALB/c mice induced CTX (cyclooxygenase-thromboxane A2 synthetase) immunomodulatory models	20, 40, and 80 mg/kg	[62]

ND: no data.

## 3. The Role of Sulfate of Fucoidan in Bioactivity

The correlation between the degree of sulfation in fucoidan and its bioactivity is significant and well documented across multiple studies [63,64,65,66,67,68]. The degree of sulfation varies among different algae species, and numerous studies have shown that sulfate content plays a pivotal role in modulating fucoidan’s biological efficacy (Table 2). Research indicates that sulfate content is a crucial factor in determining fucoidan’s biological activities, particularly its antioxidant and anticoagulant properties. These effects, however, are influenced by the structural characteristics of fucoidan. The sulfate groups in fucoidan are primarily attached to fucose residues, often at the C-2 and C-4 positions [69]. The sulfation pattern directly impacts its bioactivity, with higher sulfate content imparting a stronger negative charge, which enhances its binding interactions with specific proteins.

The impact of sulfation on fucoidan’s bioactivity also extends to its anticoagulant properties. A minimum charge density of 0.5 sulfate groups per sugar unit is required for fucoidan to exhibit effective procoagulant activity in factor VIII/factor IX-deficient plasma [70]. For instance, polysaccharides extracted from *Sargassum aquifolium* (Phaeophyceae, Fucales), though with an unspecified sulfate content, have shown anticoagulant and antitumor activities in vitro, specifically against human epithelial carcinoma cell lines (HepG2, A549, and HBL-100) [43]. In contrast, polysaccharides derived from *Sargassum horneri* (Phaeophyceae, Fucales), with a defined sulfate content of 18.47%, have demonstrated significant anti-inflammatory properties, inhibiting inflammatory responses in RAW 264.7 cells with an IC50 value of 87.12 μg/mL, suggesting that higher sulfation enhances their anti-inflammatory potential [22].

Other algae species such as *Undaria pinnatifida* (Phaeophyceae, Laminariales) and *Fucus distichus* subsp. *evanescens* exhibit notable anticancer properties, with sulfate contents of 29.14% and 28%, respectively [56,60]. Polysaccharides from *Undaria pinnatifida* have shown anticancer efficacy in vivo in Sprague Dawley rats, with concentrations of fucoidans ranging from 100 to 300 mg/kg body weight. Similarly, polysaccharides from *Fucus distichus* subsp. *evanescens* demonstrate anticancer activity in human malignant melanoma cell lines at fucoidan concentrations of 100–400 μg/mL. These findings underscore the importance of sulfate groups in enhancing the anticancer effects of algae-derived polysaccharides. The degree of sulfation and the position of sulfate groups on the fucoidan backbone are directly related to its potential activities [71]. For instance, 4O-sulfation has been shown to significantly contribute to the anticancer activity of fucoidans, and when 4O-sulfation was removed using an endo-sulfatase, the resulting desulfated fucoidans exhibited reduced inhibition of colony formation in cancer cells [72].

Polysaccharides from *Fucus serratus* and *Fucus distichus* subsp. *evanescens* have been shown to promote bone formation and vascularization, with sulfate contents of 21.54% and 46.88%, respectively. The bioactivity of these polysaccharides has been observed in human outgrowth endothelial cells (OECs) and mesenchymal stem cells (MSCs) at concentrations of 100 μg/mL. The higher sulfate content in *Fucus distichus* subsp. *evanescens* appears to enhance its effectiveness in promoting bone formation and vascularization, suggesting a correlation between sulfate content and biological activity in tissue regeneration [48].

Polysaccharides from *Saccharina latissima*, with a sulfate content of 14.3%, have exhibited immunostimulatory and hypocholesterolemic activities in vitro. The presence of sulfate groups is believed to play a significant role in modulating the immune response [49,50]. Similarly, *Cladosiphon okamuranus* polysaccharides, containing 17.6% sulfate, have been effective in alleviating atopic dermatitis symptoms through immunomodulation, as demonstrated in RAW 264.7 cell models and BALB/c mice. These studies further support the link between sulfation and immunomodulatory activities in algae-derived polysaccharides [52].

Antioxidant and antiviral properties are also closely associated with sulfate content. Polysaccharides from *Macrocystis pyrifera*, containing 26.0 ± 0.6% sulfate, have demonstrated antioxidant activity in zebrafish models, providing protective effects against oxidative stress [45]. The ratio of sulfate content to fucose has been proposed as an effective indicator of antioxidant activity in fucoidan samples. Higher sulfate content enhances fucoidan’s effectiveness as an antioxidant, likely due to its increased capacity to scavenge free radicals. On the antiviral front, *Stoechospermum polypodioides*, with a sulfate content of 13%, exhibits potent antiviral activity in vitro, with studies on Vero cells showing an effective concentration (EC50) of 3.5 ± 0.63 μg/mL, highlighting the role of sulfate groups in boosting antiviral efficacy [51].

Polysaccharides from *Saccharina japonica*, with sulfate contents of 28.7 ± 2.6%, 24.7 ± 0.9%, and 28.7 ± 2.6% in different fucoidan fractions, have shown both antibacterial and anti-SARS-CoV-2 activities. These findings suggest that varying levels of sulfation influence their antiviral and antibacterial properties [53]. Additionally, *Ascophyllum nodosum* polysaccharides, with a sulfate content of 22.6 ± 0.8%, have demonstrated promising results in attenuating SARS-CoV-2 infection in Syrian hamster models, further supporting the potential of sulfated polysaccharides as therapeutic agents against viral infections [44].

Interestingly, the relationship between sulfation and bioactivity is not always straightforward. In some cases, increasing sulfate content enhances certain activities while diminishing others. For instance, when the sulfate content of low-molecular-mass fucoidan was increased from 18.7% to 32.1%, its antioxidant and anti-inflammatory activities improved, but its anti-lipogenesis activity decreased [73].

The degree of sulfation in algae-derived polysaccharides is a key determinant of their bioactivity, with higher sulfate content often correlating with increased biological efficacy. These findings highlight the need for further research to optimize sulfation processes and elucidate the underlying mechanisms by which sulfate groups enhance the bioactive properties of these compounds. Such research could pave the way for the development of novel therapeutic agents based on sulfated polysaccharides from algae.

**Table 2:** Influence of sulfate group content and monosaccharide distribution in brown algae fucoidan on its bioactivity.

Algae Source	Sulfate (%)	Fuc (%)	Glc (%)	Xyl (%)	Man (%)	GlcA (%)	Rha (%)	Gal (%)	UAs (%)	Bioactivity	Experimental Methodologies	Experiment Model	Concentration	Reference
*Sargassum aquifolium*	ND	9.2	ND	2.2	2	2.2	ND	8.5	12.6	Anticoagulant and antitumor activities	In vitro	Human cancer cell lines HepG2 (hepatocellular carcinoma), LU-1 (lung adenocarcinoma), and RD (rhabdomiosarcoma)	ND	[43]
*Sargassum plagiophyllum*	F1: 9.8 F2: 21.9 F3: 15.1	F1: 55.5 F2: 71.1 F3: 69.1	ND	F1: 4.5 F2: 2.5 F3: 1.9	F1: 15.7 F2: 11.2 F3: 9.9	ND	ND	F1: 22.9 F2: 13.5 F3: 12.2	F1: 22.9 F2: 12.6 F3: 16.3	Anticancer activity	In vitro	HepG2, A549, and HBL-100	IC50 F1: 800 μg/mL F2: 600 μg/mL F3: 700 μg/mL	[54]
*Sargassum horneri*	18.47	36.86	ND	7.38	11.27	ND	5.23	30.09	ND	Anti-inflammatory activity	In vitro	RAW 264.7 cells	IC50 = 87.12 μg/mL	[22]
	41	85	10	ND	ND	ND	ND	5	ND	Antitumor	In vitro	The DLD-1 (ATCC # CCL-221™) human colon carcinoma cell line	200 ug/mL	[55]
*Sargassum crassifolium*	27.5	54.36	ND	1.49	0.6	ND	ND	43.55	7.6	Immunomodulatory activity	In vitro	Bone marrow cells from C3H/HeJ female mice	3 μg/mL	[74]
*Stoechospermum polypodioides*	13	96	ND	2	ND	ND	ND	2	ND	Antiviral activity	In vitro	Vero cells by a virus plaque reduction assay	EC50 (μg/mL): 3.55 ± 0.63	[51]
*Sargassum polycystum*	22.35 ± 0.23	46.8	11.5	13.2	5.6	ND	8.6	14.3	ND	Antioxidant activity, anticancer activity	In vitro	MCF-7 cells	25, 50, 75, 100, 125, and 150 μg/mL	[59]
*Sargassum siliquosum*	6.01 ± 0.53	47.13 ± 0.47	8.53 ± 4.13	9.07 ± 0.38	6.97 ± 2.93	ND	3.47 ± 0.12	24.83 ± 0.74	ND	Antioxidant activity, anti-inflammatory activity	In vitro	RAW 264.7 cell	0.25–1 μg/mL	[15]
*Fucus serratus*	21.54	76.2	ND	6.5	ND	11.2	ND	3.3	ND	Bone formation and vascularization	In vitro	Human outgrowth endothelial cells (OECs), mesenchymal stem cells (MSCs)	100 μg/mL	[48]
*Fucus distichus* subsp. *evanescens*	46.88	76.7	ND	9.8	ND	ND	ND	5.7	ND					
	28	87.1	1.3	1.8	4.4	2	ND	1.6	ND	Anticancer activity	In vitro	The SK-MEL-5 (ATCC # HTB-70), SK-MEL-28 (ATCC # HTB-72) human malignant melanoma cell lines	100–400 μg/mL	[60]
*Saccharina latissima*	14.3	59.1 ± 2.7	3.2 ± 1.6	3.0 ± 1.1	2.0 ± 0.6	ND	ND	20.8 ± 4.2	12.0 ± 2.1	Immunostimulatory, hypocholesterolemic activities	In vitro	Lymphocyte Stimulatory Activity (spleen cell of BALB/c mice) Assessment of Hypocholesterolemic Effect (in vitro intestinal model)	25, 100, 250 μg/mL	[49,50]
*Cladosiphon okamuranus*	17.6	52.7	ND	ND	ND	ND	ND	ND	18	Alleviates atopic dermatitis symptoms through immunomodulation	In vitro In vivo	RAW 264.7 cells; Atopic dermatitis (AD) model_Male BALB/c mice aged 6 weeks old	31.25, 62.5, 125, 250, 500, and 1000 μg/mL	[52]
*Sargassum fusiforme*	17.6 ± 0.36	46.32	ND	ND	24	ND	1.17	27.36	ND	Anti-inflammatory	In vitro In vivo	RAW 264.7 macrophages; zebrafish embryo	25, 50, and 100 μg/mL	[47]
*Macrocystis pyrifera*	26.0 ± 0.6	ND	ND	ND	ND	ND	ND	ND	ND	Antioxidant activities	In vivo	Zebrafish	0.625 mM	[45]
*Padina boergesenii*	17.72 ± 0.25	43.1 ± 0.23	11.6 ± 0.10	14.2 ± 0.12	ND	ND	ND	17.3 ± 0.17	9.43 ± 0.17	Antioxidant and anticancer	In vitro	Human cervical carcinoma cells (HeLa cell line)	20, 40, and 60 μg/mL	[61]
*Fucus vesiculosus*	30.8 ± 4.2	ND	ND	ND	ND	ND	ND	ND	ND	Antioxidant activities	In vivo	Zebrafish	0.625 mM	[45]
	9.9 ± 2.9	90.4 ± 2.0	ND	2.4 ± 0.7	ND	ND	ND	3.3 ± 0.7	3.8 ± 0.7	Inhibition of inflammatory response	In vitro	RAW 264.7 macrophages	0.1 μg/mL	[46]
*Saccharina japonica*	Fucoidan: 28.7 ± 2.6 Dfuc1: 24.7 ± 0.9 Dfuc2: 23.3 ± 1.0	Fucoidan: 69.14 Dfuc1: 69.84 Dfuc2: 58.55	ND	ND	Fucoidan: 12.86 Dfuc1: 13.69 Dfuc2: 20.73	ND	ND	Fucoidan: 18.00 Dfuc1: 16.43 Dfuc2: 20.73	Fucoidan: 16.2 ± 0.4 Dfuc1: 15.0 ± 0.6 Dfuc2: 15.5 ± 1.0	Antibacterial activity and anti SARS-CoV-2	In vitro	Bacteria including Staphylococcus aureus ATCC6538, Listeria monocytogenes ATCC19115, Escherichia coli ATCC25922, Shigella flexneri CMCC51574, Salmonella typhimurium ATCC14028, and Vibrio parahaemolyticus CGMCC1.1614	50, 25, 12.5, 6.25, and 0 μg/mL	[53]
*Undaria pinnatifida*	29.14	27.15	19.34	ND	ND	ND	ND	53.51	3.21	Anticancer activity	In vivo	Sprague Dawley rats	100, 200, and 300 mg/kg of body weight	[56]
	25.1 ± 1.4	ND	ND	ND	ND	ND	ND	ND	ND	Antioxidant activities	In vivo	Zebrafish	0.625 mM	[45]
	29.9 ± 0.5	92.7	ND	ND	1.5	1	ND	33.1	ND	Attenuation of SARS-CoV-2 infection	In vitro In vivo	Caco-2-Nint cells, a producer cell line expressing the SARS-CoV-2 N protein via lentiviral transduction	7.8, 15.6, 31.3, 62.5, 125, 500, and 1000 μg/mL	[44]
*Ascophyllum nodosum*	22.6 ± 0.8	56.9	ND	ND	1.5	1	ND	7.4	5.3 ± 0.1			Four-week-old female specific-pathogen-free (SPF) Syrian hamsters	Orally gavaged with high dose (Hd; 200 mg/day/kg body weight) or low dose (Ld; 100 mg/day/kg body weight	
*Ishige okamurae*	27.6	59	ND	10	9	ND	8	11	ND	Effect on recovery from immunosuppression	In vivo	BALB/c mice induced CTX (cyclooxygenase-thromboxane A2 synthetase) immunomodulatory models	20, 40, and 80 mg/kg	[62]

ND, no data; Fuc, fucose; Glc, glucose; Xyl, xylose; Man, mannose; GlcA, glucuronic acid; Rha, rhamnose; Gal, galactose; UAs: uronic acids.

## 4. The Role of Molecular Weight of Fucoidan in Bioactivity

Fucoidan and its molecular weight play a pivotal role in determining various bioactivities [75,76,77,78]. Generally, higher-molecular-weight fucoidan tends to exhibit greater bioactivity; however, the degree of sulfation can influence this effect. The relationship between molecular weight and bioactivity is not always consistent. As molecular weight increases, the number of potential sulfation sites also rises, which can lead to stronger biological activities in vivo, such as antiviral effects by binding to pathogens or viruses and blocking their activity. Additionally, lower-molecular-weight fucoidans likely enhance absorption and interaction with cellular targets, contributing to significant biological effects, including modulation of cellular signaling pathways involved in cell proliferation and immune regulation, as well as direct anticancer and antioxidant activities. Research has shown that the molecular weight of algae-derived fucoidan significantly affects its solubility, absorption, distribution, and interactions with biological targets, ultimately modulating its therapeutic potential (Table 3). *Undaria pinnatifida* contains fucoidan with a molecular weight of 97.9 kDa, demonstrating anticancer activity in vitro, particularly in studies using Sprague Dawley rats at dosages of 100, 200, and 300 mg/kg of body weight [56]. This molecular weight may reflect an optimal balance between bioavailability and biological efficacy. Fucoidans from *Fucus distichus* subsp. *evanescens*, with a molecular weight of 60 kDa, have shown strong anticancer activity in human malignant melanoma cell lines, and their relatively low molecular weight likely facilitates enhanced cellular uptake and more effective interaction with cancer cells, demonstrating higher cytotoxicity and greater inhibitory activity of cell transformation, thus resulting in enhanced anticarcinogenicity [60]. In contrast, *Sargassum horneri* contains fucoidan with a low-to-high molecular weight range of 20–140 kDa, exhibiting significant antitumor activity in human colon carcinoma cells [55].

Conversely, fucoidans with higher molecular weights, such as those derived from Fucus serratus, which have a molecular weight of 272 kDa, have been shown to promote bone formation and vascularization, as demonstrated in human outgrowth endothelial cells (OECs) and mesenchymal stem cells (MSCs) [48]. The high molecular weight of these fucoidans may provide structural advantages that are crucial for scaffolding in bone and vascular applications, enhancing binding capabilities and prolonging retention in biological systems, leading to sustained therapeutic effects. In addition, fucoidans from *Fucus distichus* subsp. *evanescens*, with a molecular weight of 84 kDa, have similarly been found to promote bone formation and vascularization, with a molecular weight offering a balance between bioactivity and tissue penetration [48].

In terms of immune modulation, fucoidans from *Saccharina latissima*, with a molecular weight of 137 kDa, have shown immunostimulatory activity in assays featuring spleen cell derived from BALB/c mice, suggesting that this molecular weight may be optimal for triggering immune responses [49,50]. On the other hand, *Cladosiphon okamuranus* contains fucoidans with a molecular weight of 49.8 kDa, which have been effective in alleviating atopic dermatitis symptoms through immunomodulation in both BALB/c mice and RAW 264.7 cells [52]. It has been found that the lower molecular weight of these fucoidans may allow for more efficient absorption and interaction with immune cells, thereby enhancing their immunomodulatory effects.

Fucoidans from *Macrocystis pyrifera*, with a low molecular weight of 70.4 kDa, have demonstrated antioxidant activity in zebrafish models [45]. The molecular weight of these fucoidans likely optimizes solubility and cellular uptake, contributing to their antioxidant properties. Similarly, fucoidans from *Stoechospermum polypodioides*, with a molecular weight of 40 kDa, have exhibited potent antiviral activity [51]. Fucoidans with lower molecular weights are often more effective in antiviral applications, as their size facilitates better penetration of viral envelopes and interaction with viral proteins. In the case of *Saccharina japonica*, fucoidans with varying molecular weights, including 90.8 kDa, have demonstrated antibacterial and anti-SARS-CoV-2 activity [53].

Similarly, fucoidans from *Ascophyllum nodosum*, with a molecular weight of 124.3 kDa, have shown promise in attenuating SARS-CoV-2 infection in Syrian hamsters, suggesting that higher molecular weights may contribute to the stability and duration of antiviral effects [44]. The relationship between molecular weight and bioactivity is evident across various algae species, with relatively low-molecular-weight fucoidans generally showing better absorption, solubility, and bioavailability, which enhances their biological effects, such as their anti-inflammatory and antiviral activities. Conversely, higher-molecular-weight fucoidans may offer structural advantages and prolonged therapeutic effects, making them suitable for applications such as bone formation and immune modulation. Based on these studies, future research should emphasize that the specific effects of fucoidan can vary depending on the particular bioactivity being investigated, highlighting the importance of adjusting fucoidan’s molecular weight to suit the desired applications [79].

**Table 3:** Relationship between molecular weight of brown algae source and bioactivity.

Algae Source	Molecular Weight (kDa)	Bioactivity	Experimental Methodologies	Experiment Model	Concentration	Reference
*Sargassum aquifolium*	ND	Anticoagulant and antitumor activities	In vitro	Human cancer cell lines HepG2 (hepatocellular carcinoma), LU-1 (lung adenocarcinoma), and RD (rhabdomiosarcoma)	ND	[43]
*Sargassum plagiophyllum*	F1: 20 F2: 35 F3: 30	Anticancer activity	In vitro	HepG2, A549, and HBL-100	ND	[54]
*Sargassum horneri*	20–140	Antitumor	In vitro	The DLD-1 (ATCC # CCL-221™) human colon carcinoma cell line	200 μg/mL	[55]
	30	Anti-inflammatory activity	In vitro	RAW 264.7 cells	IC50 = 87.12 μg/mL	[22]
*Dictyopteris divaricata*	58.05	Antioxidant and immunomodulatory activities	In vivo	RAW 264.7 murine macrophages	ND	[57]
*Sargassum crassifolium*	230	Immunomodulatory activity	In vitro	Bone marrow cells from C3H/HeJ female mice	3 μg/mL	[74]
*Stoechospermum polypodioides*	40	Antiviral activity	In vitro	Vero cells by a virus plaque reduction assay	EC50 (μg/mL): 3.55 ± 0.63	[51]
*Sargassum polycystum*	ND	Antioxidant activity, anticancer activity	In vitro	MCF-7 cells	25, 50, 75, 100, 125, and 150 μg/mL	[59]
*Sargassum siliquosum*	ND	Antioxidant activity, anti-inflammatory activity	In vitro	RAW 264.7 cell	0.25–1 μg/mL	[15]
*Fucus serratus*	272	Bone formation and vascularization	In vitro	Human outgrowth endothelial cells (OECs), mesenchymal stem cells (MSCs)	100 μg/mL	[48]
*Fucus evanescens*	84					
	60	Anticancer activity	In vitro	The SK-MEL-5 (ATCC # HTB-70), SK-MEL-28 (ATCC # HTB-72) human malignant melanoma cell lines	100–400 μg/mL	[60]
*Saccharina latissima*	137	Immunostimulatory, hypocholesterolemic activities	In vitro	Lymphocyte Stimulatory Activity (spleen cell of BALB/c mice) Assessment of Hypocholesterolemic Effect (in vitro intestinal model)	25, 100, and 250 μg/mL	[49,50]
*Cladosiphon okamuranus*	49.8	Alleviates atopic dermatitis symptoms through immunomodulation	In vitro In vivo	RAW 264.7 cells; atopic dermatitis (AD) model_Male BALB/c mice aged 6 weeks old	31.25, 62.5, 125, 250, 500, and 1000 μg/mL	[52]
*Sargassum fusiforme*	60–150	Anti-inflammatory	In vitro In vivo	RAW 264.7 macrophages; zebrafish embryo	25, 50, and 100 μg/mL	[47]
*Macrocystis pyrifera*	70.4	Antioxidant activities	In vivo	Zebrafish	0.625 mM	[45]
*Padina boergesenii*	224	Antioxidant and anticancer	In vitro	Human cervical carcinoma cells (HeLa cell line)	20, 40, and 60 μg/mL	[61]
*Fucus vesiculosus*	97.7	Antioxidant activities	In vivo	Zebrafish	0.625 mM	[45]
	70	Inhibition of inflammatory response	In vitro	RAW 264.7 macrophages	0.1 μg/mL	[46]
*Saccharina japonica*	Fucoidan: 90.8 Dfuc1: 19.2 Dfuc2: 5.5	Antibacterial activity and anti SARS-CoV-2	In vitro	Bacteria including Staphylococcus aureus ATCC6538, Listeria monocytogenes ATCC19115, Escherichia coli ATCC25922, Shigella flexneri CMCC51574, Salmonella typhimurium ATCC14028, and Vibrio parahaemolyticus CGMCC1.1614	50, 25, 12.5, 6.25, and 0 μg/mL	[53]
*Undaria pinnatifida*	97.9	Anticancer activity	In vivo	Sprague Dawley rats	100, 200, and 300 mg/kg of body weight	[56]
	168.5	Antioxidant activities	In vivo	Zebrafish	0.625 mM	[45]
	141.7	Attenuation of SARS-CoV-2 infection	In vitro In vivo	Caco-2-Nint cells, a producer cell line expressing the SARS-CoV-2 N protein via lentiviral transduction	7.8, 15.6, 31.3, 62.5, 125, 500, and 1000 μg/mL	[44]
*Ascophyllum nodosum*	124.3			Four-week-old female specific-pathogen-free (SPF) Syrian hamsters	Orally gavaged with high dose (Hd; 200 mg/day/kg body weight) or low dose (Ld; 100 mg/day/kg body weight)	
	1.4–40	Antioxidant activity	In vitro	Scavenging activity on DPPH radical		[80]
*Ishige okamurae*	12.9	Effect on recovery from immunosuppression	In vivo	BALB/c mice induced CTX (cyclooxygenase-thromboxane A2 synthetase) immunomodulatory models	20, 40, and 80 mg/kg	[62]

ND: no data.

## 5. The Role of Glycosidic Linkage of Fucoidan in Bioactivity

Fucoidan’s bioactivity is significantly influenced by its diverse glycosidic bonds, which are crucial for determining its biological functions (Table 4). Typically, fucoidan’s backbone is composed of α-L-fucopyranose residues linked mainly through (1→3) and (1→4) glycosidic linkages [81,82]. These linkages not only determine the structural conformation of fucoidan but also affect its solubility, interactions with biological targets, and overall bioactivity. The configuration, position, and type of these glycosidic bonds greatly impact fucoidan’s antioxidant, anti-inflammatory, anticancer, antiviral, and immunomodulatory properties.

In particular, the ratio of (1→3) to (1→4) linkages influences how fucoidan interacts with different enzymes and cell surface receptors. For example, fucoidans with a higher proportion of (1→3) linkages tend to exhibit enhanced antioxidant and antiviral activities, likely due to their more flexible and accessible structure. Meanwhile, (1→4)-linked fucoidans are associated with stronger anti-inflammatory and anticancer properties, possibly because of their more rigid conformation, which may enhance interactions with immune cells and tumor microenvironments [83].

Regarding fucoidans from *Sargassum aquifolium*, they contain 2-linked α-d-Manp and 4-linked β-d-GlcAp glycosidic bonds, which have been associated with anticoagulant and antitumor activities in vitro [43]. These effects were observed in cells derived from human cancer cell lines such as the HePG2, human cervical carcinoma, and RD cell lines. The presence of these specific glycosidic linkages likely enhances the fucoidans’ interactions with key cell surface receptors involved in coagulation and tumor suppression, contributing to their bioactivity. *Undaria pinnatifida* contains fucoidans with β-d-Galp, α-type glycosidic linkages, which have demonstrated notable anticancer effects in vitro, particularly in studies with Sprague Dawley rats [56]. The specific glycosidic bonds, in this case, contribute to structural conformation that facilitates binding to cancer cell receptors such as selectins, thereby triggering apoptotic and anti-proliferative pathways, which are key to fucoidan’s anticancer properties [84,85,86].

Fucoidans from *Padina borgeseni* contain (1→4)-l-fucose, (1→6)-β-d-galactose, and β-d-mannuronic acid linkages, which contribute to their antioxidant and anticancer activities [61]. The arrangement of these glycosidic bonds plays a critical role in free radical scavenging, reducing oxidative stress in cells and enhancing the fucoidans’ therapeutic potential. Additionally, fucoidan from *Stoechospermum polypodioides*, with [(1→4) and (1→3)-linked α-l-fucopyranosyl] glycosidic bonds, has exhibited strong antiviral activity in vitro against Vero cells. These linkages, combined with sulfate groups, likely enhance binding affinity to viral components, effectively inhibiting viral replication at an EC50 of 3.5 ± 0.63 μg/mL [51,87,88].

Moreover, a specific structure from *Sargassum horneri* was identified as a branched polysaccharide, with a backbone consisting of repeating units of →3-α-L-Fucp(2SO_3_^−^)-1→4-α-L-Fucp(2,3SO_3_^−^)-1→ and side chains including α-L-Fucp-1→2-α-L-Fucp-1→ or α-L-Fucp-1→3-α-L-Fucp(4SO_3_^−^)-1→ attached at C4 [55]. The high molecular weight and abundance of α-l-Fucp-1→3-α-l-Fucp(4SO_3_^−^)-1→ side chains exhibited the most potent anticancer and radiosensitizing effects on the colony formation of DLD-1 cells.

Fucoidans from *Saccharinajaponica* contain a variety of glycosidic bonds, including α-(1→3)-linked fucose residues, which have been linked to both antibacterial and anti-SARS-CoV-2 activity [53]. The complexity and diversity of these bonds provide multiple interaction sites with bacterial and viral proteins, amplifying the bioactivity of the fucoidan [14,89]. Furthermore, *Undaria pinnatifida* fucoidan has (α1→4)-linked l-fucopyranose glycosidic bonds, which play a crucial role in attenuating SARS-CoV-2 infection [44]. The structural conformation driven by these bonds likely interferes with viral entry or replication, making these fucoidans promising antiviral candidates [90,91].

Moreover, the position of glycosidic linkages can influence the conformational entropy of oligosaccharides, which, in turn, affects their recognition, processing, and overall properties [92]. This suggests that specific linkage patterns in fucoidan may contribute to its diverse bioactivities, including its anticoagulant, antioxidant, and anti-inflammatory effects [81]. For instance, fucoidan with alternating (1→3) and (1→4) linkages has shown stronger inhibitory effects on α-D-glucosidase, which is relevant for its anti-diabetic activity [82].

Another study on fucoidan from *Sargassum binderi* suggested a main structure of →3)fuc-2-OSO_3_^−^ (1→3)fuc-2-OSO_3_^−^ (1→ [93], which indicates its safety for food product applications. Although experiments on biological activity were not conducted alongside structural elucidation, some fucoidans, such as those from the sea cucumber *Pearsonothuria graeffei*, exhibit a tetrasaccharide repeating unit with a backbone of [→3Fuc (2S, 4S) α1→3Fucα1→3Fuc (4S) α1→3Fuc]n [94]. In the case of fucoidans from *Cladosiphon okamuranus*, the backbone consists of α-1,3-linked fucopyranoside units with fucose branching at C-2 [95].

Interestingly, glycosidic linkages can vary within the same fucoidan molecule. For instance, fucoidan from Ascophyllum nodosum features a highly branched core region with predominantly α-(1→3)-linked fucosyl residues and some α-(1→4) linkages, with branch points at position 2 of the →3-linked internal residues [96]. This structural complexity contributes to the diverse biological activities of fucoidans, suggesting that variations in the structural features may be significant for their various biological properties.

The glycosidic bond connections in algae-derived fucoidan play a crucial role in determining its biological activity. Linkages such as (1→3) and (1→4)-linked α-fucopyranose or β-D-Galp-(1→4) significantly influence fucoidan’s structural conformation and its ability to interact with biological targets, thereby enhancing its bioactivity. A deeper understanding of glycosidic bonds in fucoidan can provide essential insights for developing novel therapeutic agents. Future research should focus on further elucidating the precise structures of these linkages and their mechanisms of action to maximize the therapeutic potential of algae-derived fucoidan in clinical applications [82,97].

## 6. Advancements in Analytical Techniques: Exploring the Diverse Structure of Fucoidan

Initially, fucoidan was believed to consist solely of sulfated fucose. This understanding persisted until the discovery of fucoidan in *Macrocystis pyrifera* in the early 1980s, which revealed the presence of not only fucose but also other monosaccharides such as galactose and xylose, challenging the earlier, simplified view of fucoidan’s composition [98]. Subsequent studies confirmed that fucoidan is much more structurally diverse than previously thought, with its composition varying depending on the source and species. Research demonstrated that the monosaccharide composition of fucoidan could include a range of polysaccharides and that this variability contributed to its functional diversity [99]. To analyze this complexity, researchers employed various analytical techniques, such as acetylation analysis, high-performance liquid chromatography (HPLC), and gas–liquid chromatography (GLC), which allowed for precise identification and quantification of the monosaccharides present in fucoidan, enhancing our understanding of fucoidan’s intricate structure [98,100].

**Figure 1:**
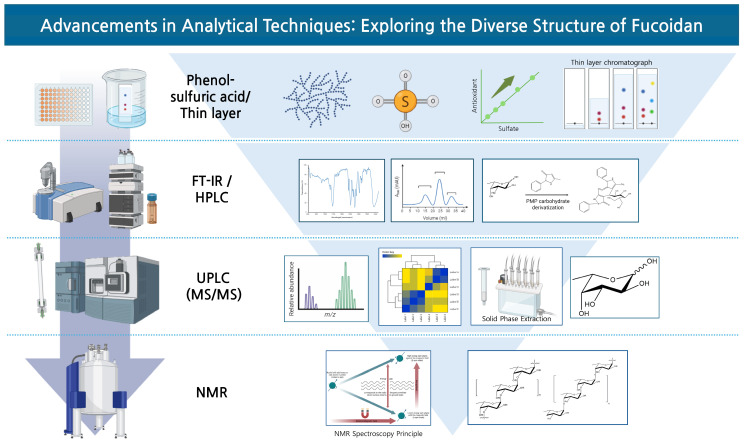
Analytical techniques used to explore the complex structure of the sulfated polysaccharide fucoidan, including the phenol–sulfuric acid method/thin-layer chromatography (TLC), Fourier transform infrared (FT-IR) spectroscopy, high-performance liquid chromatography (HPLC), ultra-performance liquid chromatography–tandem mass spectrometry (UPLC-MS/MS), and nuclear magnetic resonance (NMR) spectroscopy. Phenol–sulfuric acid and TLC methods are applied for the quantification of primary carbohydrates and analysis of sulfate groups. The increase in sulfate groups is positively correlated with the increase in antioxidant capacity, and the spots of different colors represent unique compounds or chemical components of the sample mixture. FT-IR spectroscopy and HPLC are used for functional group identification and monosaccharide composition analysis, while UPLC-MS/MS provides detailed structural insights through the mass-to-charge ratio (*m*/*z*) distribution, cluster analysis, and solid-phase extraction. Finally, NMR spectroscopy provides a comprehensive understanding of the backbone structure and branching pattern of the polysaccharide. Each technique provides unique information, allowing for a deeper understanding of the diverse and complex structural properties of fucoidan.

The significant advances in analytical technologies over recent decades have greatly enhanced the ability to elucidate the complex structure of fucoidan. (Figure 1) In the past, fucoidan’s heterogeneous and highly branched structure made detailed structural analysis challenging. However, the development of high-resolution mass spectrometry (HR-MS) has provided researchers with the ability to accurately determine the molecular weights of fucoidan’s polysaccharide residues and map the bonding patterns between them, offering a clearer view of its structural complexity [101]. In addition, nuclear magnetic resonance (NMR) spectroscopy has significantly complemented the elucidation of fucoidan’s complex structure [58,102]. It has played a crucial role in determining the primary structure of fucoidan, including its repeating units and sulfation patterns, enabling researchers to construct a more comprehensive three-dimensional model of fucoidan. For example, enzymatic degradation, methylation analysis, and NMR spectroscopy have been used together to clarify the primary structure of fucoidan from *Holothuria tubulosa* [58]. Additionally, high-performance size-exclusion chromatography combined with multiple-angle laser light scattering and viscometry can provide information on chain conformation and molecular weight [58].

Chromatographic techniques such as HPLC and GC have further aided in separating and analyzing fucoidan’s components, particularly when coupled with advanced methods like mass spectrometry or methylation analysis. These techniques, along with enzymatic degradation methods, have made it possible to break down fucoidan into smaller oligosaccharides, simplifying the study of its structural motifs and facilitating a deeper understanding of its diverse monosaccharide makeup.

Hydrophilic interaction liquid chromatography (HILIC) coupled with electrospray mass spectrometry (ESI-FTMS) and high-energy collision-induced dissociation (HCD-MS/MS), along with 2D NMR spectroscopy analysis, has been successfully employed to map fucoidan oligosaccharides [103]. These techniques provide detailed information on the sequence and sulfation patterns of oligosaccharides. ESI-MS has been used to determine the chemical structure of fucoidan, while small-angle X-ray scattering (SAXS) has also been used to confirm bonding arrangements and spatial architecture in crystallized segments of fucoidan, further contributing to the understanding of its structure [104]. ESI-MS can reveal the backbone composition and branching patterns, while SAXS provides insights into the molecular-level conformation. Spectroscopic methods such as Fourier-transform infrared (FT-IR) spectroscopy and Raman spectroscopy, combined with chemometrics, have been developed for determining the purity of extracted fucoidan [102,105,106]. FT-IR spectroscopy is useful for confirming the presence of fucoidan and identifying its structural characteristics. This technique can detect changes in functional groups and is particularly helpful in analyzing the effects of various treatments on fucoidan structure.

Additionally, advances in bioinformatics have allowed for more efficient processing and interpretation of the complex data generated by these techniques, enabling researchers to visualize and comprehend the full structural diversity of fucoidan in unprecedented detail. X-ray crystallography, though traditionally difficult to apply to large polysaccharides, has also been used to confirm bonding arrangements and spatial architecture in crystallized segments of fucoidan, further contributing to the understanding of its structure.

As these technologies have advanced, it has become clear that the chemical composition of fucoidan is not only more complex but also more variable than initially assumed. Fucoidan’s structure differs significantly depending on its source, particularly among different species of brown algae. Broadly, fucoidans have been classified into two structural types: Type I, which consists of 1,3-α-L-Fucp units with sulfate groups at the O-2 and O-4 positions, and Type II, which features alternating 1,3- and 1,4-α-L-Fucp units with sulfate groups at the O-2, O-3, and O-4 positions [107]. Recent studies have uncovered additional structural variations, including different sulfate substitution patterns, molecular weights, branching configurations, and polysaccharide residues, although they generally retain a backbone like Type I and Type II fucoidans [108]. These variations in structure are significant because they lead to different biological effects, even though these compounds are all categorized under the comprehensive term “fucoidan” [109]. Thus, the advancement of analytical technologies has not only made it easier to analyze fucoidan’s structure but has also revealed its remarkable structural diversity, providing new opportunities to explore its biological and pharmacological potentials.

## 7. Conclusions

Fucoidan has long been studied for its diverse range of biological activities, traditionally attributed to the presence and distribution of sulfate groups. However, accumulating evidence now suggests that the monosaccharide composition, specifically the types, ratios, and distribution of monosaccharides such as fucose, galactose, and mannose, plays an equally critical, if not more significant, role in modulating its bioactivity.

The factor of a lower molecular weight enhances bioavailability by enabling easier absorption and cellular uptake, while the factor of a higher molecular weight prolongs therapeutic effects due to facilitation of slower degradation and extended circulation in the body. The sulfate content and specific binding positions of sulfate groups are critical in modulating fucoidan’s bioactivity, as they influence its ability to interact with proteins and cell receptors. The factor of a higher sulfate content increases the negative charge of fucoidan, enhancing electrostatic interactions with positively charged biological molecules, which is crucial for its anticoagulant, antiviral, and anti-inflammatory effects. Similarly, the composition of polysaccharides, including the types and ratios of monosaccharides, affects the structural conformation of fucoidan, impacting its binding affinity to receptors and its ability to trigger specific biological responses. These combined factors, including polysaccharide composition, sulfate content, the binding positions of sulfate groups, and molecular weight, work synergistically to determine fucoidan’s therapeutic potential by influencing its stability, receptor binding, and overall bioactivity. Additionally, external factors such as extraction methods can lead to variations in fucoidan’s chemical characteristics, even within the same species of algae. This variability may result in differences in biological activity, further emphasizing the need for more systematic research. This highlights the need for a comprehensive understanding of fucoidan’s characteristic–function relationship, rather than focusing solely on sulfate groups, to explain the reasons behind fucoidan’s bioactivity.

Future research should prioritize the in-depth structural characterization of fucoidan by focusing on optimizing methods for extraction, purification, and analysis to better control polysaccharide composition, molecular weight distribution, and sulfation binding patterns. Additionally, investigating how molecular weight and structural configuration influence cleavage, absorption, receptor binding, the production of secondary metabolites, and subsequent biological interactions is crucial. By advancing the understanding of these factors, including receptor interactions and bioavailability, fucoidan’s full therapeutic potential can be realized. These insights are essential for developing precise fucoidan-based therapies, leading to the comprehensive application of fucoidan.

There are not many sulfated biopolymers like fucoidan, but some sulfated polysaccharides have been found in marine organisms. With the recent advancements in the structural analysis of marine-derived polysaccharides, research on the diverse properties of these sulfated polysaccharides is expected to progress rapidly. While studies on the correlation between the characteristics and biological activities of sulfated polysaccharides other than fucoidan are still in the early stages, it is anticipated that more comprehensive data on the relationships between the monosaccharide composition, abundance, and biological activity of marine-derived sulfated polysaccharides will be published and utilized in the future. This will likely lead to a clearer understanding of the biological mechanisms and structure–function relationships of not only fucoidan but also other sulfated polysaccharides.

## Figures and Tables

**Table 4:** Role of glycosidic bond structures in brown algae in determining its bioactivity.

Algae Source	Glycosidic Bond Connection	Bioactivity	Experimental Methodologies	Experiment Model	Concentration	Reference
*Sargassum aquifolium*	[2-linked α-d-Manp and 4-linked β-d-GlcpA]	Anticoagulant and antitumor activities	In vitro	Human cancer cell lines HepG2 (hepatocellular carcinoma), LU-1 (lung adenocarcinoma), and RD (rhabdomiosarcoma)	ND	[43]
*Sargassum horneri*	[α-l-Fucp-1→3-α-l-Fucp(4SO_3_^−^)-1→]	Antitumor	In vitro	The DLD-1 (ATCC # CCL-221™) human colon carcinoma cell line	200 μg/mL	[55]
*Fucus distichus* subsp. *evanescens*	[3)-α-L-Fucp-(2SO_3_^−^)-(1→4)-α-L-Fucp-(2,3SO_3_^−^)-(1→]	Anticancer activity	In vitro	The SK-MEL-5 (ATCC # HTB-70), SK-MEL-28 (ATCC # HTB-72) human malignant melanoma cell lines	100–400 μg/mL	[60]
*Sargassum crassifolium*	[3)-α-L-Fucp-(1→3)-α-L-Fucp (SO_3_^−^)-(1→4)-α-L-Fucp-(SO_3_^−^)-(1→]	Immunomodulatory activity	In vitro	Bone marrow cells from C3H/HeJ female mice	3 μg/mL	[74]
*Sargassum polycystum*	[3)-α-L-Fucp-(1→3)-α-L-Fucp-(1→]	Antioxidant activity, anticancer activity	In vitro	MCF-7 cells	25, 50, 75, 100, 125, and 150 μg/ml	[59]
*Sargassum siliquosum*	[3)-α-L-Fucp-(2SO_3_^−^)-(1→4)-α-L-Fucp-(1→]	Antioxidant activity, anti-inflammatory activity	In vitro	RAW 264.7 cell	0.25–1 μg/mL	[15]
*Padina boergesenii*	(1–4)-L fucose, (1–6) β-D galactose, α and β-D Manncronic acid	Antioxidant and anticancer	In vitro	Human cervical carcinoma cells (HeLa cell line)	20, 40, and 60 μg/mL	[61]
*Saccharina* *japonica*	A:→3)-α-l-Fucp(2,4S)-(1→ B:→3)-α-l-Fucp(2S)-( C:→3)-α-l-Fucp-(1→ D:→4)-β-d-Manp(1→ E:→6)-β-d-Galp(1→	Antibacterial activity and anti SARS-CoV-2	In vitro	Bacteria including Staphylococcus aureus ATCC6538, Listeria monocytogenes ATCC19115, Escherichia coli ATCC25922, Shigella flexneri CMCC51574, Salmonella typhimurium ATCC14028, and Vibrio parahaemolyticus CGMCC1.1614	50, 25, 12.5, 6.25, and 0 μg/mL	[53]
*Undaria pinnatifida*	[β-D-Galp, α-type glycosidic linkages]	Anticancer activity	In vivo	Sprague Dawley rats	100, 200, and 300 mg/kg of body weight	[56]
	α(1,4)-linked L-fucopyranose	Attenuation of SARS-CoV-2 infection	In vitro In vivo	Caco-2-Nint cells, a producer cell line expressing the SARS-CoV-2 N protein via lentiviral transduction	7.8, 15.6, 31.3, 62.5, 125, 500, 1000 μg/mL	[44]
*Ascophyllum nodosum*	[(1→3) and (1→4) linked α-l-fucopyranose]			Four-week-old female specific-pathogen-free (SPF) Syrian hamsters	Orally gavaged with high dose (Hd; 200 mg/day/kg body weight) or low dose (Ld; 100 mg/day/kg body weight)	
*Ishige okamurae*	[→3)-α-l-Fucp-(1→, →4)-α-l-Fucp-(1→, →6)-β-d-Galp-(1→ and →3)-β-d-Galp-(1 → residues with sulfate groups at C-2/C-4 the of (1→3)-α-l-Fucp and C-6 the of (1→3)-β-d-Galp]	Effect on recovery from immunosuppression	In vivo	BALB/c mice induced CTX (cyclooxygenase-thromboxane A2 synthetase) immunomodulatory models	20, 40, and 80 mg/kg	[62]
*Stoechospermum polypodioides*	[(1→4)- and (1→3)-linked-α-l-fucopyranosyl]	Antiviral activity	In vitro	Vero cells by a virus plaque reduction assay	EC50 (μg/mL): 3.55 ± 0.63	[51]

ND: no data.

## Data Availability

No new data were created or analyzed in this study. Data sharing is not applicable to this article.

## Supplementary Materials

The following supporting information can be downloaded at: https://www.mdpi.com/article/10.3390/ijms252111771/s1.
