# Supplementary material for: Systematic Characteristics of Fucoidan: Intriguing Features for New Pharmacological Interventions
Source: Int J Mol Sci. 2024 Nov 1;25(21):11771. doi: 10.3390/ijms252111771 (PMC11546589; doi:10.3390/ijms252111771)
Supplement: Supplementary file 1 [file ijms-25-11771-s001.zip › ijms-3268918-supplementary.pdf]

Supplementary Table S1. Analysis of structural factors influencing the bioactivity of brown algae fucoidan: effects of monosaccharide composition, sulfate group content, molecular weight, and glycosidic bond structures

Algae source	Monosaccharide composition	sulfate (%)	Fuc (%)	Glc (%)	Xyl (%)	Man (%)	GlcA (%)	Rha (%)	Gal (%)	UA (%)	Molecular Weight (kDa)	Glycosidic bond connection	Bioactivity	Experimental Methodologies	Experiment model	Concentration	Reference
Sargassum aquifolium	Fuccose, Galactose, Mannose, Glucuronic acid, Xylose	ND	9.2	ND	2.2	2	2.2	ND	8.5	12.6	ND	[2-linked α-d-Manp and 4-linked β-d-GlcpA]	Anticoagulant and anti-tumor activities	In vitro	Human cancer cell lines HepG2 (hepatocellular carcinoma), LU-1 (lung adenocarcinoma), and RD (rhabdomiosarcoma)	ND	[43]
Sargassum plagiophyllum	Fucose, Galactose, Xylose, Mannose	F1 9.8 F2: 21.9 F3: 15.1	F1 55.5 F2: 71.1 F3: 69.1	ND	F1 4.5 F2: 2.5 F3: 1.9	F1 15.7 F2: 11.2 F3: 9.9	ND	ND	F1 22.9 F2: 13.5 F3: 12.2	F1 22.9 F2: 12.6 F3: 16.3	F1 20 F2: 35 F3: 30	[α-l-Fucp-1→3-α-l-Fucp(4SO3−)-1→]	Anticancer activity	In vitro	HepG2, A549 and HBL-100	IC50 F1 800 μg/mL F2: 600 μg/mL F3: 700 μg/mL	[54]
Sargassum horneri	Fucose, Galactose, Mannose, Xylose, Rhamnose	18.47	36.86	ND	7.38	11.27	ND	5.23	30.09	ND	20-140	ND	Anti-Inflammatory activity	In vitro	RAW 264.7 cells	IC50 = 87.12 μg/mL	[22]
	Fucose, Galactose, Mannose, Glucuronic acid	41	85	10	ND	ND	ND	ND	5	ND	30	[3)-α-L-Fucp-(2SO3–)-(1→4)-α-L-Fucp-(2,3SO3–)-(1→]	Anti-tumor	In vitro	The DLD-1 (ATCC # CCL-221™) human colon carcinoma cell line	200 ug/mL	[55]
Dictyopteris divaricata	Fucose, Xylose, Mannose, Glucose, Galactose	ND	ND	ND	ND	ND	ND	ND	ND	ND	58.05	ND	Antioxidant and immunomodulatory activities	In vivo	RAW264.7 murine macrophages	ND	[57]
Sargassum crassifolium	Fucose, Galactose	27.5	54.36	ND	1.49	0.6	ND	ND	43.55	7.6	230	[3)-α-L-Fucp-(1→3)-α-L-Fucp (SO3–)-(1→4)-α-L-Fucp-(SO3–)-(1→]	immunomodulatory activity	In vitro	Bone marrow cells from C3H/HeJ female mice	3 μg/mL	[74]
Stoechospermum polypodioides	Fucose, Xylose, Mannose, Galactose, Glucose, Galacturonic acid	13	96	ND	2	ND	ND	ND	2	ND	40	[(1 → 4)- and (1 → 3)-linked-α-l-fucopyranosyl]	antiviral activity	In vitro	Vero cells by a virus plaque reduction assay	EC50 (μg/ml): 3.55 ± 0.63	[51]

Sargassum polycystum	Fucose, Xylose, Mannose, Galactose, Glucose, Rhamnose	22.35 ± 0.23	46.8	11.5	13.2	5.6	ND	8.6	14.3	ND	ND	[3)-α-L-Fucp-(1→3)-α-L-Fucp-(1→]	antioxidant activity, anticancer activity	In vitro	MCF-7 cells	25, 50, 75, 100, 125 and 150 μg/mL	[59]
Sargassum siliquosum	Fucose, Xylose, Mannose, Galactose, Glucose, Rhamnose	6.01 ± 0.53	47.13 ± 0.47	8.53 ± 4.13	9.07 ± 0.38	6.97 ± 2.93	ND	3.47 ± 0.12	24.83 ± 0.74	ND	ND	[3)-α-L-Fucp-(2SO3−)-(1→4)-α-L-Fucp-(1→]	antioxidant activity, anti-inflammatory activity	In vitro	RAW264.7 cell	0.25–1 μg/mL	[10]
Fucus serratus	Fucose, Galactose, Glucuronic acid, Xylose	21.54	76.2	ND	6.5	ND	11.2	ND	3.3	ND	272	ND	Bone Formation and Vascularization	In vitro	human outgrowth endothelial cells (OEC), mesenchymal stem cells (MSC)	100 μg/mL	[48]
	Fucose, Xylose, Galactose	46.88	76.7	ND	9.8	ND	ND	ND	5.7	ND	84	ND					
Fucus distichus subsp. *evanescens*	Fucose, Xylose, Mannose, Galactose, Glucose, Glucuronic acid	28	87.1	1.3	1.8	4.4	2	ND	1.6	ND	60	[3)-α-L-Fucp-(2SO3−)-(1→4)-α-L-Fucp-(2,3SO3−)-(1→]	anticancer activity	In vitro	The SK-MEL-5 (ATCC # HTB-70), SK-MEL-28 (ATCC # HTB-72) human malignant melanoma cell lines	100–400 μg/mL	[60]
Saccharina latissima	Fucose, Xylose, Galactose, Mannose, Glucuronic acid, Uronic aicd	14.3	59.1 ± 2.7	3.2 ± 1.6	3.0 ± 1.1	2.0 ± 0.6	ND	ND	20.8 ± 4.2	12.0 ± 2.1	137	ND	Immunostimulatory, Hypocholesterolemic activities	In vitro	Lymphocyte Stimulatory Activity (spleen cell of BALB/c mice) Assessment of Hypocholesterolemic Effec (in vitro intestinal model)	25, 100, 250 μg/mL	[49, 50]
Cladosiphon okamuranus	Fucose, Uronic aicd	17.6	52.7	ND	ND	ND	ND	ND	ND	18	ND	ND	alleviates atopic dermatitis symptoms through immunomodulation	In vitro In vivo	RAW264.7 cells Atopic dermatitis (AD) model_Male BALB/c mice of 6 weeks' old	31.25, 62.5, 125, 250, 500, and 1000 μg/mL.	[52]
Sargassum fusiforme	Fucose, Galactose, Mannose	17.6 ± 0.36	46.32	ND	ND	24	ND	1.17	27.36	ND	60-150	ND	anti-inflammatory	In vitro In vivo	RAW 264.7 macrophages zebrafish embryo	25, 50, 100 μg/mL	[47]
Macrocystis pyrifera	Fucose, Xylose, Glucuronic acid	26.0 ± 0.6	ND	ND	ND	ND	ND	ND	ND	ND	70.4		antioxidant activities	in vivo	zebrafish	0.625 mM	[45]
Padina boergesenii	Fucose, Galactose, Glucose, Xylose	17.72 ± 0.25	43.1 ± ± 0.23	11.6 ± 0.10	14.2 ± 0.12	ND	ND	ND	17.3 ± 0.17	9.43 ± 0.17	224	(1–4)-L fucose, (1–6) β-D galactose, α and β-D Manncronic acid	antioxidant and anticancer	In vitro	human cervical carcinoma cells (HeLa)	20, 40, and 60 μg/mL	[61]

Fucus vesiculosus	Fucose, Xylose, Galactose, Mannose	30.8 ± 4.2	ND	ND	ND	ND	ND	ND	ND	ND	97.7	ND	antioxidant activities	in vivo	zebrafish	0.625 mM	[45]
	Fucose, Xylose, Galactose, Uronic aicds	9.9 ± 2.9	90.4 ± 2.0	ND	2.4 ± 0.7	ND	ND	ND	3.3 ± 0.7	3.8 ± 0.7	70	ND	Inhibition of inflammatory response	In vitro	RAW 264.7 Macrophages	0.1 μg/mL	[46]
Saccharina japonica	Fucose, Mannose, Glucose	Fucoidan: 28.7 ± 2.6 Dfuc1: 24.7 ± 0.9 Dfuc2: 23.3 ± 1.0	Fucoidan: 69.14 Dfuc1: 69.84 Dfuc2: 58.55	ND	ND	Fucoidan: 12.86 Dfuc1: 13.69 Dfuc2: 20.73	ND	ND	Fucoidan: 18.00 Dfuc1: 16.43 Dfuc2: 20.73	Fucoidan: 16.2 ± 0.4 Dfuc1: 15.0 ± 0.6 Dfuc2: 15.5 ± 1.0	Fucoidan: 90.8 Dfuc1: 19.2 Dfuc2: 5.5	A:→3)-α-l-Fucp(2,4S)-(1→ B:→3)-α-l-Fucp(2S)-(C:→3)-α-l-Fucp-(1→ D:→4)-β-d-Manp(1 → E:→6)-β-d-Galp(1→	antibacterial activity and anti SARS CoV 2	In vitro	Bacteria including Staphylococcus aureus ATCC6538, Listeria monocytogenes ATCC19115, Escheric hia coli ATCC25922, Shigella flexneri CMCC51574, Salmonella typhimurium ATCC14028, and Vibrio parahaemolyticus CGMCC 1.1614	50, 25, 12.5,6.25,0 μg/mL	[53]
	Fucose, Glucose, Galactose	29.14	27.15	19.34	ND	ND	ND	ND	53.51	3.21	97.9	[β-D-Galp, α-type glycosidic linkages]	Anticancer activity	In vivo	Sprague-Dawley rats	100, 200 and 300 mg/kg of body weight	[56]
Undaria pinnatifida	Fucose, Glucuronic Acid, Galactose, Mannose	25.1 ± 1.4	ND	ND	ND	ND	ND	ND	ND	ND	168.5	ND	antioxidant activities	in vivo	zebrafish	0.625 mM	[45]
	Fucose, Glucuronic acid, Galactose, Mannose	29.9 ± 0.5	92.7	ND	ND	1.5	1	ND	33.1	ND	141.7	α(1,4)-linked L-fucopyranose	attenuate SARS-CoV-2 infection	In vitro	Caco-2-Nint cells, a producer cell line expressing the SARS-CoV-2 N protein via lentiviral transduction	7.8, 15.6, 31.3, 62.5, 125, 500, 1000 μg/mL	[44]
Ascophyllum nodosum	Fucose, Galactose, Mannose, Glucuronic acid, Uronic aicd	22.6 ± 0.8	56.9	ND	ND	1.5	1	ND	7.4	5.3 ± 0.1	124.3	[(1 → 3) and (1 → 4) linked α-l-fucopyranose]		In vivo	Four-week-old female specific-pathogen-free (SPF) Syrian hamsters	orally gavaged with high-dose (Hd; 200 mg/day/kg body weight) or low-dose (Ld; 100 mg/day/kg body weight	

Ishige okamurae	Fucose, Galactos, Glucose, Xylose	27.6	59	ND	10	9	ND	8	11	ND	12.9	[→3)-α-l-Fucp-(1→, →4)-α-l-Fucp-(1→, →6)-β-d-Galp-(1 → and →3)-β-d-Galp-(1 → residues with sulfate groups at C-2/C-4 the of (1 → 3)-α-l-Fucp and C-6 the of (1 → 3)-β-d-Galp]	effect on recovery from immunosuppressio n	In vivo	BALB/c mice induced CTX (cyclooxygenase-thromboxane A2 synthetase) Immunomodulatory models	20, 40, 80 mg/kg	[62]

* ND, No data; Fuc, Fucose; Glc, Glucose; Xyl, Xylose; Man, Mannose; GlcA, Glucuronic acid; Rha, Rhamnose; Gal, Galactose; UA: Uronic acids.
